# Effective relax acquisition: a novel approach to classify relaxed state in alpha band EEG-based transformation

**DOI:** 10.1186/s40708-024-00225-y

**Published:** 2024-05-13

**Authors:** Diah Risqiwati, Adhi Dharma Wibawa, Evi Septiana Pane, Eko Mulyanto Yuniarno, Wardah Rahmatul Islamiyah, Mauridhi Hery Purnomo

**Affiliations:** 1 Departement of Electrical Engineering, Institut Teknologi Sepuluh Nopember, Keputih, Surabaya, 60111 Indonesia; 2 Departement of Computer Engineering, Institut Teknologi Sepuluh Nopember, Keputih, Surabaya, 60111 Indonesia; 3 Medical Technology, Institut Teknologi Sepuluh Nopember, Keputih, Surabaya, 60111 Indonesia; 4 Departement of Informatics, Universitas Muhammadiyah Malang, Tlogomas, Malang, 65144 Indonesia; 5 Industrial Training and Education of Surabaya, Ministry of Industry RI, Gayungan, Surabaya, 60235 Indonesia; 6 Neurology Department, Faculty of Medicine, Universitas Airlangga, Gubeng, Surabaya, 60131 Indonesia

**Keywords:** Relaxed state analysis, EEG, EEG dimensional reduction, Transformation signal, Alpha sub-band

## Abstract

A relaxed state is essential for effective hypnotherapy, a crucial component of mental health treatments. During hypnotherapy sessions, neurologists rely on the patient’s relaxed state to introduce positive suggestions. While EEG is a widely recognized method for detecting human emotions, analyzing EEG data presents challenges due to its multi-channel, multi-band nature, leading to high-dimensional data. Furthermore, determining the onset of relaxation remains challenging for neurologists. This paper presents the Effective Relax Acquisition (ERA) method designed to identify the beginning of a relaxed state. ERA employs sub-band sampling within the Alpha band for the frequency domain and segments the data into four-period groups for the time domain analysis. Data enhancement strategies include using Window Length (WL) and Overlapping Shifting Windows (OSW) scenarios. Dimensionality reduction is achieved through Principal Component Analysis (PCA) by prioritizing the most significant eigenvector values. Our experimental results indicate that the relaxed state is predominantly observable in the high Alpha sub-band, particularly within the fourth period group. The ERA demonstrates high accuracy with a WL of 3 s and OSW of 0.25 s using the KNN classifier (90.63%). These findings validate the effectiveness of ERA in accurately identifying relaxed states while managing the complexity of EEG data.

**Graphical abstract:**

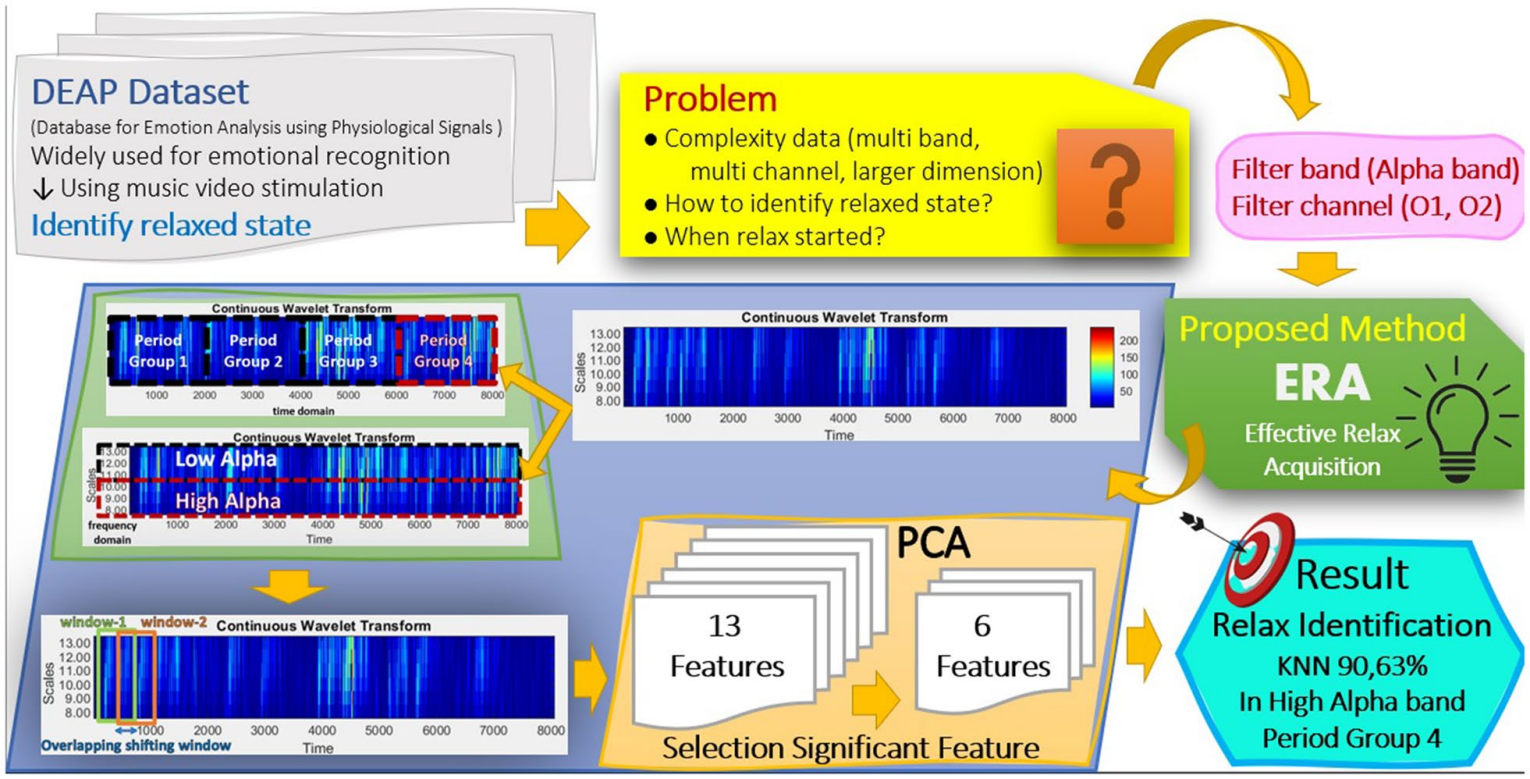

## Introduction

Mental disorders also referred to as mental illnesses, encompass behavioural or psychological conditions that may cause personal distress or impairments in specific areas of functioning. These conditions arise from various factors, including genetic predispositions, biological influences, brain injuries, or prolonged exposure to stress. As part of the rehabilitation process, neurologists often employ hypnosis to address these disorders. However, the effectiveness of hypnosis relies heavily on the physical and emotional state of the patient. Patients must achieve a relaxed state to optimize the effects of hypnosis. Neurologists usually evaluate the patient’s state of relaxation through the observation of their Electroencephalogram (EEG) signals. Recognizing a relaxed state holds significant importance in various mental health interventions, such as therapeutic interventions for mental illnesses, rehabilitation programs, insomnia treatment, and pain reduction in conditions like gastrointestinal disorders and cancer [1, 2].

The relaxed state pattern can manifest through various physical characteristics in patients, including blood pressure, heart rate, oxygen consumption, muscular tension, and breathing patterns [3]. However, depending solely on these physical traits to observe the relaxed state can pose challenges because each patient may display distinct physical characteristics, potentially resulting in unclear analysis outcomes. To resolve this matter, neurologists employ electroencephalogram (EEG) signals for a more precise understanding of the patient’s psychological condition. Examining EEG data enables neurologists to conduct a comprehensive evaluation of the emotional and attentional characteristics exhibited by each patient. By positioning electrodes on the scalp, neurologists can directly monitor brain activity, enabling a more thorough assessment of patients’ psychological conditions [4].

EEG data is collected by placing electrode sensors directly on the patient’s scalp. These sensors can capture data with varying specifications, ranging from 1 to 256 channels. Each channel comprises five bands: delta, theta, alpha, beta, and gamma [5, 6]. However, using more sensors increases the complexity of the data due to the relationship between the number of channels and their corresponding band components. The complexity of this data presents challenges, especially considering the need to extract multiple features from EEG data and categorize them into time, frequency, and time-frequency domains [7]. Consequently, effective management of EEG data necessitates appropriate feature selection, emphasizing the importance of dimensionality reduction.

The EEG signal consists of five distinct bands, each defined by its specific frequency range. The delta band, ranging from 0.5 Hz to 3 Hz, is evident during deep sleep. The theta band, ranging from 4 Hz to 7 Hz, indicates drowsiness or emotional stress. The alpha band, ranging from 8 Hz to 13 Hz, signifies wakefulness or a relaxed state. The beta band, covering frequencies from 14 Hz to 39 Hz, is associated with concentration or deep thought. Finally, the gamma band, ranging from 31 Hz to 100 Hz, is prominent during complex activities involving visual, auditory, and motoric activity.

In our experiment, we specifically concentrate on analysing the alpha band to identify relaxation patterns. Prior studies have successfully utilized the alpha band to identify similar patterns [4, 8, 9]. This pattern often manifests in the posterior area of the brain, particularly in the occipital region [10]. Furthermore, researchers have utilized the alpha band to identify developmental disorders in children related to brain maturation [11], and to analyse epileptic seizures occurring while awake or asleep [12]. Numerous researchers have explored the alpha band’s frequency or time domain to identify the relaxation pattern. Elsadek investigated the induction of relaxation by comparing individuals viewing greenery through high-rise building windows versus urban scenes. The study revealed higher relative alpha power in the AF3 channel when participants viewed green spaces compared to urban settings. Measurements from channels AF4, O1, and O2 also yielded similar results [13]. Kusumandari explored the effects of methadone medication on alpha and theta amplitudes, inducing relaxation by instructing subjects to close and open their eyes. The experiment observed an increase in alpha-band activity after methadone consumption. Specifically, alpha band amplitude increased when subjects closed their eyes and decreased when they opened them [9]. Unfortunately, there have not been any researchers studying the specific moment when the relaxed state appears. As knowing this would be very helpful in the field of neurology, especially in the practice of hypnotherapy, we focus our research to fill this gap.

To achieve this objective, we propose a detailed analysis of the alpha sub-band within the full alpha band in both the frequency and time domains. As we know, Alpha band spans from 8–13 Hz, and it can be separated into two sub-bands with the High Alpha at 11–13 Hz and the Low Alpha at 8–10 Hz [14, 15].

Xu observes the relaxed condition from 235 students, who were stimulated by fragments of Slavic folklore. From this observation Xu finds that there is a shifting in the Low Alpha behavior: the border of the low alpha power value is increasing which indicates the relaxed state [16]. In addition, Babiloni finds that the low alpha is dominant during a relaxed condition while the high alpha in the opposite behavior [17]. Meanwhile, Kawashima utilizes multi modal combination between brain and heart rate to ensure the relaxed state. Kawashima creates simulation through music listing stimulation with resting break. During the resting time, Kawashima measures the fatigue and sleepiness level from each participant. He finds that there are oscillations in the alpha band, and by breaking down into Low Alpha and High Alpha he found correlation with physiological states and mental functions [18]. However, Teplan’s findings contradict previous research. Teplan observed a decrease in low alpha power during the relaxed state. This discrepancy may be attributed to scenarios where participants had to close their eyes to achieve relaxation [19]. Thus, the stimulation scenario may influence the differentiation of Low Alpha and High Alpha brain activity in individuals. Feature extraction plays a crucial role in recognizing the relaxed state as part of emotion, as there are numerous hidden pieces of information that are needed to obtain detailed emotional insights. Janke’s study emphasizes the role of emotion by categorizing emotional features into the time domain, frequency domain, and time-frequency domains. The study revealed that each emotion exhibits unique characteristics, requiring specific feature extraction [20]. Suppiah studies brain signals using frequency domain approach. Suppiah investigates brain signals using a frequency domain approach, observing the relaxed state through power band features via Power Spectral Density (PSD). The findings suggest that normalized alpha PSD values are highly reliable indicators of the relaxed state, with accuracy rates ranging from 97% to 99% [21]. Tyas concentrates on observing activity in the theta, alpha, and beta bands in the occipital region as a method to classify mental fatigue. This experiment utilizes time domain features such as standard deviation and mean absolute, demonstrating a significant correlation between these features in O1 channel [22].

Additionally, relaxed state classification can also be extracted through wavelet signal transformation. This method involves converting data from the time and frequency domain into the time-frequency domain, aiming to obtain more detailed features for specific emotional classifications. Lestari utilizes a combination of time-frequency domains by employing the CWT Alpha approach to investigate the relaxed state through normalization and data segmentation scenarios. In this experiment, she employs two characteristics: entropy and energy, derived from the wavelet transform coefficient. The results of her experiment indicate that processing EEG data through normalization is unsuitable as it affects the model performance [4]. Another researcher employs data augmentation by extending the data into multiple time segmentations with different overlapping shift window strategies, which proves suitable for a machine learning approach [23, 24]. Pane analyses the correlation between two time-frequency domain features using the Higuchi algorithm and power spectral density (PSD). This involves sampling data with a time duration of 9 s, followed by emotional classification using Ripper, J.48, and SVM classifiers, which are effective in distinguishing between relaxed and sad emotions [23]. Candra observes wavelet features with different time segment scenarios and finds that shorter time segment wavelet entropy increases classification accuracy [25]. On the other hand, longer signal segments might introduce data inconsistency because they could potentially mix with other information, resulting in ambiguity. However, it is crucial to conduct time domain segmentation with caution to prevent immature data due to inadequate information.

Classifying emotions presents a tough challenge as we strive to identify the most relevant features. However, incorporating too many features can significantly increase data complexity and computational costs [20]. Moreover, the intricacies of EEG data often result in irrelevant and redundant attributes, leading to suboptimal classification performance [26]. To address this challenge, it is crucial to decrease both the dimensions of data and features. Principal Component Analysis (PCA) is widely employed for this purpose, as it effectively reduces dimensions while preserving a high level of variance [27, 28]. For instance, Omuya devised a hybrid filter model that combines PCA with Information Gain for feature selection. This innovative approach aims to select the most pertinent feature set, enhancing classification performance while minimizing computational expenses [26].

In this experiment, our focus is on exploring the alpha band to discern the characteristics of the relaxed state through signal transformation. By conducting an in-depth analysis of each sub-band, we aim to determine which sub-band has the most significant influence on the relaxed state. For the time-domain analysis, the data is segmented into four-period groups to identify the initial onset of the relaxed state condition. To improve the prediction accuracy of the relaxed state, we employ a unique approach that combines time segmentation with time-frequency domain analysis. This method will enable neurologists to accurately identify the precise moment when an individual enters a relaxed state.

Therefore, we propose an Effective Relax Acquisition (ERA) method for recognizing the relaxed state using EEG data. This study contributes to classifying the relaxed state in the following ways:

1. The ERA identifies the alpha band as a characteristic of the relaxed state by subdividing it into two sub-bands (High and Low Alpha sub-bands).
2. The ERA suggests relaxing acquisition in the time domain by dividing EEG data into 4-period groups to pinpoint the timing of relaxed conditions.
3. The ERA utilizes PCA to reduce the number of features by composing the most significant Principal Component (PC).

The structure of the paper is as follows: Section 2 offers a detailed explanation and formulation of the proposed method. Section 3 presents the experimental results. Afterward, Sect. 4 explores a discussion on the ERA and incorporates a comparative analysis with related work. Lastly, Sect. 5 present the conclusions drawn from this study.

## Proposed method

### Block diagram of ERA

**Fig. 1:**
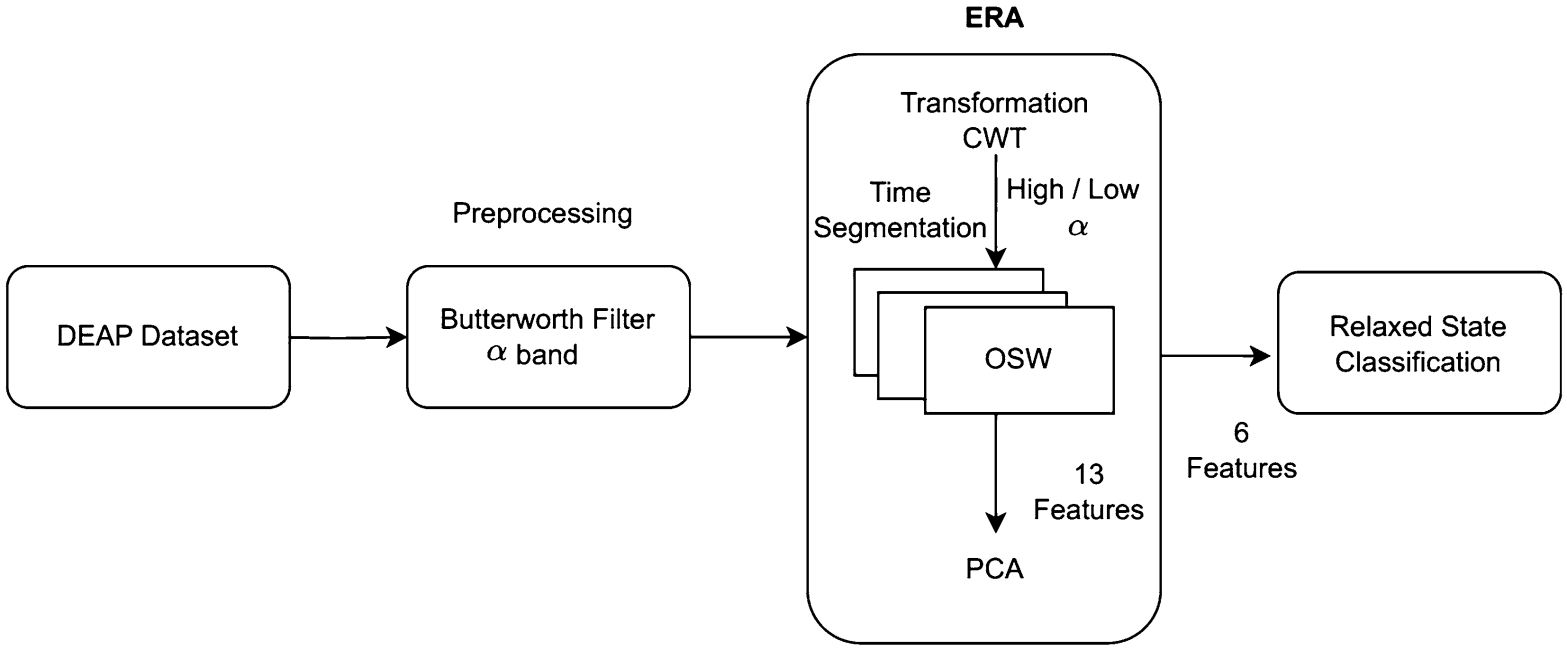
The proposed method of ERA

Our proposed method, ERA, is depicted in the block diagram shown in Fig. 1. This diagram consists of three stages: The first stage involves preprocessing, including the application of a Butterworth band-pass filter. The second stage is the ERA, which encompasses signal transformation, feature extraction, and dimension reduction. The third stage focuses on classification using multiple classifiers.

In this experiment, we utilize a publicly available database for emotion analysis using physiological signals (DEAP). The DEAP dataset is widely recognized and utilized for emotion recognition tasks [24]. The DEAP dataset was recorded using 48 channels at a sampling rate of 512 Hz (down-sampled to 128 Hz) [29]. These channels comprise a combination of 32 EEG channels and 12 physiological signals, with three unused channels and one particular channel used for data status [30]. The signals were collected from a total of 32 participants, comprising 15 females and 17 males, aged between 19 and 37 years. Each EEG data has a consistent length of 63 s, separated into two segments: the first 3 s acting as baseline signals (neutral state), while the subsequent 60 s simulate stimulation by presenting music videos.

**Fig. 2:**
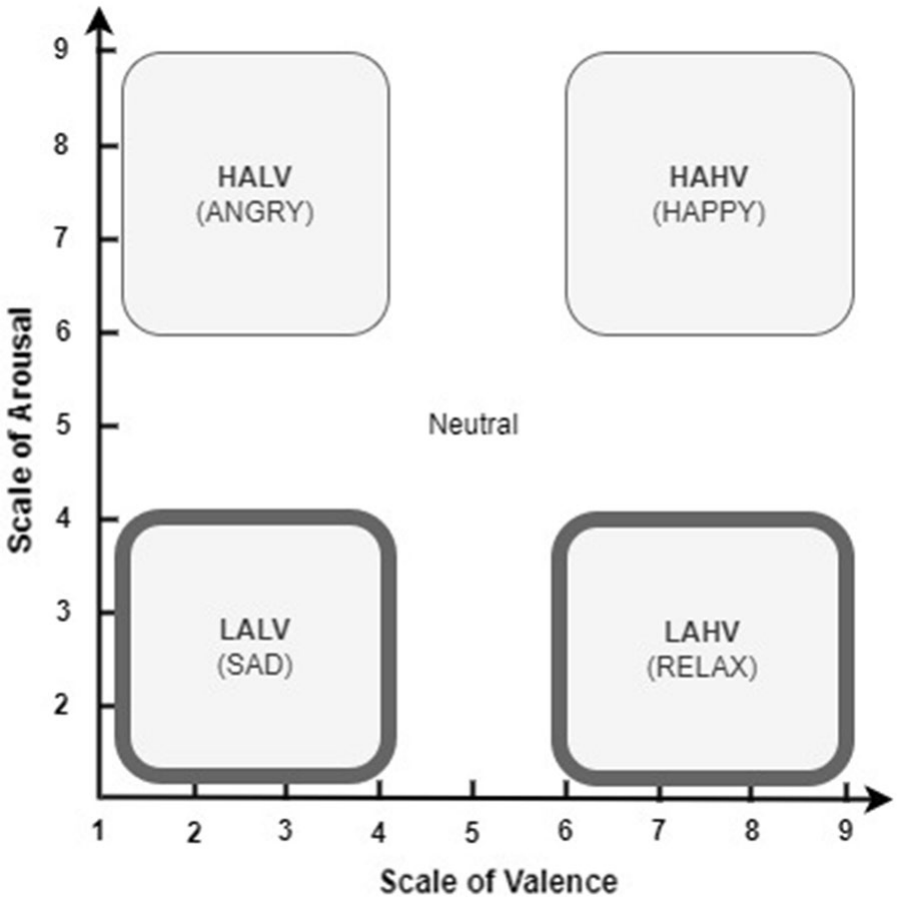
Bi-dimensional valence arousal emotional representation

We used Russell’s Circumplex model with two dimensions: valence and arousal. Figure  2 shows that valence refers to the positive degree of emotion. In contrast, arousal refers to the intensity of the emotion. Emotional response can be measured by a 9-point scale, from 1 (indicating low) to 9 (indicating high). By following these scales, we are mapping the arousal and valence scale in a two-dimensional emotional model. By using the DEVA scale as shown in Fig. 2, we are mapping low arousal by a scale of 1–4, while the high arousal scale ranges from 6–9. The degree of valence also has the same specifications as the degree of arousal [31]. This two-dimensional valence-arousal diagram, illustrated in Fig. 2, comprises four emotion quadrants: High Arousal High Valence (HAHV) representing happiness, High Arousal Low Valence (HALV) representing anger, Low Arousal High Valence (LAHV) representing relaxation, and Low Arousal Low Valence (LALV) representing sadness. During the experiment, we excluded four subjects (S13, S20, S21, and S22) as they did not exhibit a relaxed response. Pane conducted a detailed analysis of the relationship between the LAHV quadrant (relaxed state) and the other three emotional quadrants (sad, angry, and happy) and found that the best classification accuracy for relaxation occurred when comparing the LAHV vs. LALV quadrant. Based on this research, we further investigate the LAHV vs. LALV comparison [23].

Previous studies have concluded that a person with a relaxed state has a more dominant alpha band than the other bands. The alpha band can indicate that a person is relax, awake and not in a state of concentration [3, 27, 32]. Therefore, in the preprocessing stage, we carried out a Butterworth bandpass filter in the selected band signal. This bandpass filter is performed on the alpha band signal in the frequency range of 8–13 Hz. Furthermore, this study used only O1 and O2 channels at the occipital cortex (O1 and O2 channels placement) as the most relevant channels to the alpha band among all 32 channels [3, 33].

Data samples generated by the ERA are used for classification purposes. Classification was performed using several machine learning approaches, such as Support Vector Machine (SVM), K-Nearest Neighbors (KNN), and Extreme Gradient Boosting (XGB). The SVM has effective scaling for high-dimensional feature representation and error management [34–36]. While the KNN is one of the simplest classification algorithms and part of supervised machine learning techniques. The KNN is a common algorithm based on Euclidean distance [35]. The KNN is suitable for non-linear classifiers, small data dimensions, binary classification, and data with overlapping sliding windows [37–39]. The XGB is a more effective version of the gradient boosting architecture. It includes both tree learning methods and linear model solvers. To ensure that the classification is performed on random data and to avoid over-fitting problems, we used K-fold strategy with K-fold 5.

### Effective relax acquisition (ERA)

ERA consists of four parts, transformation, feature extraction, dimension reduction, and most significant feature recommendations. We used CWT in transformation phase by convolving EEG signal with Morlet basis function. This basis function will convolve original signal into wavelet form and transform it into scale and time [4, 32, 34].

Therefore, CWT as $W(s, \tau )$ can also describe the correlation between the original signal *x*(*t*) and the wavelet basis function $\varphi _{(s, \tau )}$, where ’$*$’ is the complex conjugation as described in Eq. (1). There is an established heuristic relationship that transforms scales into frequencies. Time (*t*) and scales (*s*) or the corresponding frequency $f_s$ are related. The center frequency of the wavelet defines the relationship $f_c$ in Eq. (2). While $f_s$ is pseudo frequency, and $\Delta$ is sampling period for the scale *s*.

**Fig. 3:**
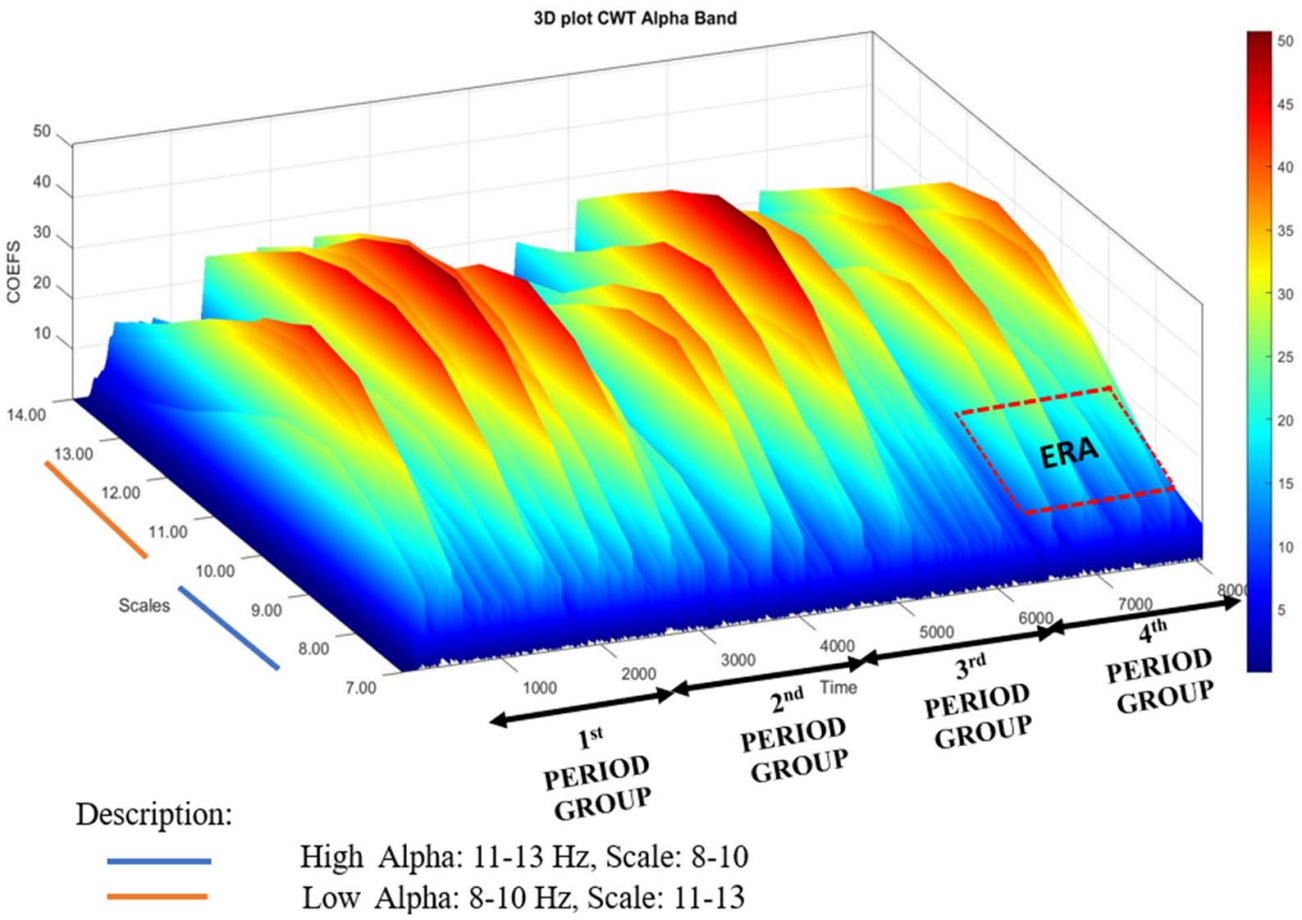
3D plot CWT on alpha band for a trial EEG signal

**Fig. 4:**
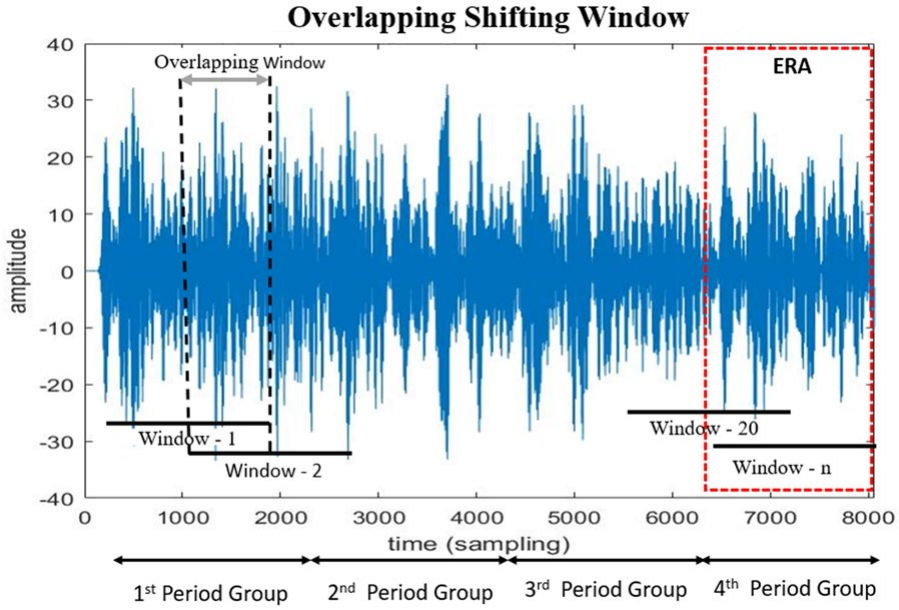
Overlapping shifting window scenarios illustration

1 $$\begin{aligned} W(s, \tau )= & {} \int _{-\infty }^{\infty } x(t)\varphi _{(s, \tau )}^* (t) \text {d}t. \end{aligned}$$

2 $$\begin{aligned} f_{s}= & {} \frac{f_{c}}{s\Delta }. \end{aligned}$$

To investigate more into the alpha band, we partitioned it into two sub-bands: Low Alpha and High Alpha. In CWT, high frequencies correspond to low scales, whereas high scales represent low frequencies. Equation (2) is used to perform wavelet decomposition scale from a frequency range of Alpha band (8–13 Hz). Sub-band Low Alpha (8–10 Hz) is based on the 11–13 wavelet scale decomposition, while the sub-band High Alpha band (11–13 Hz) is based on the 8–10 wavelet scale decomposition. Figure 3 illustrates the Alpha band decomposition into the Low Alpha and the High Alpha based on the time-frequency domain scale. Figure 3 shows alpha band in time domain. The alpha band data can be divided into 4-period groups. This period group division is defined as follows, 1st period group with sampling time 384–2304, 2nd period group with sampling time 2304–4224, 3rd period group with sampling time 4224–6144, and 4th period group with sampling time 6144–8064. By grouping the sample time into 4-period groups, then the CWT can be assumed on the Eq. (1) with the input function *x*(*t*) of length *N* vector *x*[*n*]. Thus, by entering the period group range, a CWT is obtained [38]. In Eq. (3), we can compute CWT where $\tau$ is the time shift, *i* is the lower threshold of the sampling time (for example *i *= 6.144 in 4th period group), *N* is the upper threshold of the vector *x*[*n*] (for example *N *= 8.064 in 4th period group), and the process is repeated for each scale *s*.

3 $$\begin{aligned} W_s[\tau ] = \sum _{n = i}^{N - 1} x[n]\varphi _s[\tau - n]. \end{aligned}$$

Features were extracted from sub-band High Alpha and Low Alpha over channels O1 and O2 based on multiple overlapping shifting window scenarios. Previous studies have proven that overlapping shifting windows can augment data and provide many data variations. This process is useful by making the learning process more precise [24, 40, 41]. In our experiment, we used several scenarios, with 2 window length (WL) scenarios (3 s and 4 s) and 3 overlapping shifting window (OSW) scenarios (0.5 s, 1 s, and 2 s). Figure 4 shows a scenario for a window length of 3 s and an overlapping shift window of 2 s. Therefore, the dataset specification used is shown in Table 1. And the number of overlapping shift windows (*n*-frames) can be calculated using Eq. (4).

**Table 1:** Data spesification of extracted feature

Data	Size	Description
Columns	$13 \times 3 \times 2$	Features $\times$ scales of a high or low alpha band $\times$ channels
Rows	$28 {\times } {[} 2 \,\textrm{to} \, 18{ ]} {\times } {[} 29, 57, 109 {]}$	Subjects ${\times }$ trials ${\times }$ windows length

**Table 2:** Time-frequency domain features

Feature number (F)	Feature	Formula
1	Mean	$\mu _W = \frac{1}{N}\sum _{i=1}^N W(S,\tau )_i$
2	Wavelet entropy	$W = -\sum _{j=1}^N P_{ij}\times log_2P_{ij}$
3	Mean absolute	$|\mu _W| = \biggl |\frac{1}{N} \biggl |\sum _{i=1}^N W(s,\tau )_i$
4	Standard deviation	$\sigma _W = \sqrt{\frac{\sum _{i=1}^N \bigl ( W(s,\tau )_i - \mu _W \bigr )^{2}}{N-1}}$
5	Hjorth activity	$HA = var \bigl (W(s,\tau ) \bigr )$
6	Hjorth mobility	$HM = \sqrt{\frac{var \Bigl (\frac{dW(s,\tau )_i}{d_i} \Bigr )}{var \bigl (W(s,\tau ) \bigr )}}$
7	Hjorth complexity	$HC = \frac{HM \Bigl ( \frac{W(s,\tau )_i}{d_i} \Bigr )}{HM \bigl ( W(s,\tau )_i \bigr )}$
8	Zero crossing	$n_{Zc} =\sum _{W_imin}^{W_imax}\delta _{W(s,\tau )_i}$
9	Higuchi fractal dimension	$l(k)= \frac{\sum _{m=1}^k L_m (k)}{k}$
10	Band power	$P = \lim _{T \rightarrow \infty } \frac{1}{T} \int _{0}^{T} \bigl [ W(s,\tau ) \bigr ]^2 \text {d}t$
11	Shannon entropy	$H = - \sum _{i=1}^k (P_i) log_2 (P_i)$
12	Differential entropy	$H(W) = \frac{1}{2} log (2 \pi \sigma ^2)$
13	Amplitude maximum	$A_{max} = Max \bigl ( W(s,\tau ) \bigr )$

4 $$\begin{aligned} n{\text -}frame = \frac{length\,of\, CWT}{osw - \bigl ( \frac{(wl \times f_s) - osw}{osw}\bigr )}. \end{aligned}$$

In this experiment, we extracted 13 features as shown in Table 2 from the continuous wavelet transform signal. These features are widely used in feature extraction studies in the EEG signal processing. This feature was chosen because it is suitable for measuring emotional states and is used in biological signals.

EEG data is considered high-dimensional due to its multi-channel and multi-band composition. Consequently, each set of data can yield a vast number of features. This complexity arises from the ability to process the data across the time domain, frequency domain, and time-frequency domain. Such complexity can lead to computational challenges, including increased computational cost as well as overfitting in machine learning applications. These issues commonly arise in tasks such as data classification, data mining, sentiment analysis, visualization, and compression of high-dimensional data. Addressing the dimensional problem requires minimizing information loss by simplifying correlated data dimensions while preserving the main characteristics of the original data.

This dimensional problem can be solved by using the covariance matrix *D*(*X*) to establish correlations and dependencies between each feature. Then, the covariance matrix can be decomposed with the spectral decomposition of the eigenvalue $\lambda _1,$ $\lambda _2,$ $\lambda _3,$...,$\lambda _D$ and the eigenvalue is sorted in descending order as $\lambda _1$ $\ge \lambda _2$ $\ge \lambda _3$...$\ge \lambda _D$. The sorted eigenvalue is used as a criterion to determine the principal component (PC), while *D* is the dimensional data. We can construct an eigenvector matrix *v* using the selected corresponding number of *k* eigenvectors. The *k* eigenvectors are used as row vectors to form a new space matrix that constitutes the PC. The variance of the PC can be calculated with the ratio between its corresponding eigenvalue and the total sum of all eigenvalues using Eq. (5). The result of Eq. (5) is then used to construct a PC with a cumulative contribution rate of PC that generally requires more than 95% [42, 43]. Based on these 13 features extracted from the data, a new space is created by selecting these data through Eq. (5). In order to build more effective computational cost, those 13 PCs are reduced to 6 PCs using Eq. (6). These 6 PCs form $PC_D$, where *D* is the new space created by dimension reduction. Each feature can be sorted as the most significant feature with matrix eigenvector.

5 $$\begin{aligned} variance \, PC=\, & {} \frac{\lambda _i}{\sum _{i=1}^d\lambda _i}. \end{aligned}$$

6 $$\begin{aligned} PC_D =\, & {} V_1 D(X) + V_2 D(X) + V_3 D(X) + \dots + V_D D(X). \end{aligned}$$

7 $$\begin{aligned} ERA =\, & {} min_i \Bigl ( F\bigl (W (s,\tau )(t,\Delta ) - L (t,\Delta t \bigl ) \Bigl )^2. \end{aligned}$$

Equation ( 7) is the core of the ERA calculation, this formula is used to calculate the most optimal scenario for the relaxed state classification. The most optimal scenario can be obtained with $\min _i$, where *i* is the number of windows. The function *F* is a machine learning approach to the relaxed state classification, as proposed in three machine learning methods (SVM, XGB, and KNN). While $W(s, \tau )$ is 6 PCs on the Eq. (6). Then it is compared with *L* label data (relaxed state and sad state). These two data are computed and sampled from time (*t*) through window dimension $(\Delta t)$.

The Algorithm 1 is proposed to obtain the Effective Relax Acquisition (ERA). In this algorithm, $\min _i$ is calculated from the beginning of the second window (*i*) because the first window is used as the baseline data. The output of this algorithm is the $\min _i$ window on the selected scale sub-band Low Alpha or High Alpha and occurred in which period group.

It starts with the input of the EEG data with the CWT signal preprocessing used in first six cumulative PCs. Then this data is separated into scale *s*, which separates the Alpha band into sub-bands Low Alpha, High Alpha and Alpha band itself. These data are segmented into four-period groups based on sampling time as explained in Fig. 3. Meanwhile, *i* is the number of each data frame, and it is defined as 2 because the first frame is used as the baseline data. The data are processed as an increment *i* to ensure that the calculation is performed on all data, then this process will produce $\min _i$ value for the effective relax acquisition value.

**Algorithm 1:**
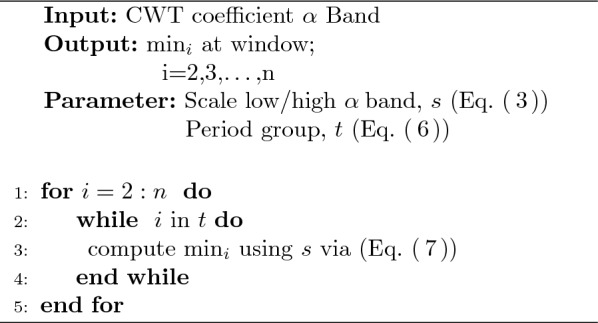
Effective relax acquisition

Furthermore, an evaluation process is carried out on the experimental results. Measuring the accuracy of the experiment is required to evaluate the effectiveness of the predictions using Eq. (8) [44]:

8 $$\begin{aligned} Accuracy (\%) = \frac{Number\ of\ correct\ predict \ sample }{number\ of\ total\ sample}. \end{aligned}$$

## Result

The aim of this experiment is to identify the relaxed state condition using the proposed ERA method. We employed a feature optimization strategy by selecting the most significant feature based on its eigenvalues. To enhance this feature, we conducted experiments using the OSW and WL strategy for data augmentation. We performed time domain analysis by organizing the data into 4-period groups to predict when the relaxed state begins.

### Feature extraction and dimensional reduction

Feature extraction is performed by following two scenarios of WL (3 s and 4 s) and three scenarios of OSW (0.5 s, 1 s and 2 s), this scenario is performed on the High Alpha, the Low Alpha and the Alpha band. Table 3 is calculated from 13 features as shown in Table 2. All of this data is calculated through the KNN classifier by following the WL and the OSW strategy. Based on the data from Table 3, we can conclude that the best scenarios can be obtained with WL = 3 s and OSW = 0.5 s. Therefore, this scenario is used as the input configuration for the PCA. The PCA is used for dimension reduction by performing variance analysis on each respective PC.

**Fig. 5:**
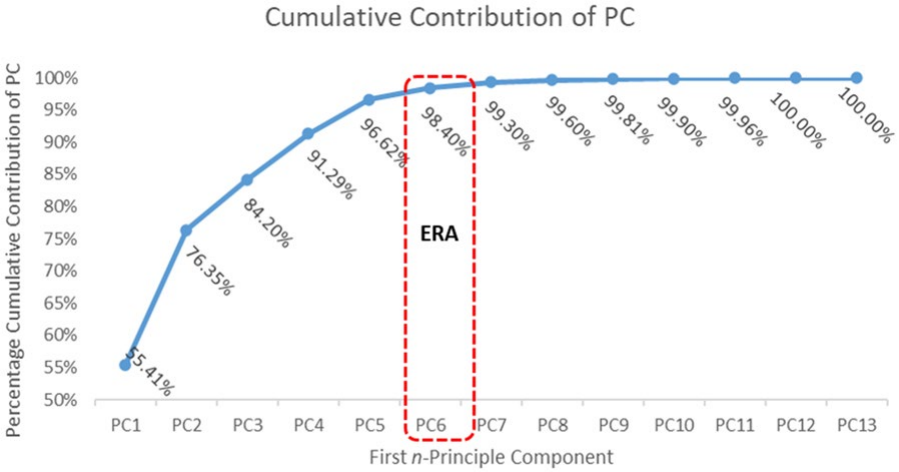
Percentage cumulative contribution from n-PCs

Figure 5 shows the percentage cumulative contribution from *n*-PC. Significant variance observations were made from 95% as the minimum threshold [42, 43] Variance PC value started significantly from PC5 with 96.62%.

The PC Data in Fig. 5 was built based on the following criteria: PC1 was built using the first principal component, while PC2 was built using the first and second principal components. Thus *n*-PC was cumulating the first PC,... nth PC. Meanwhile PC6 data is cumulating the data between 1st PC until 6th PC. By using the cumulative threshold variance, we can conclude that only PC5, PC6, PC7, PC8, PC9, PC10, PC11, PC12, and PC13 fulfill those thresholds. However, if we check carefully the differences between each PC, we can find that the most significant increment occurred between PC5 and PC6 with 1.78% increment. Meanwhile, the increment value from PC6 to PC7 is lowers to 0.9% and this trend keeps leaning until through PC13. Due to this consideration then we chose PC6 as the first six component features.

**Table 3:** Accuracy score from 13 features through the KNN classifier based on various WL and OSW strategies

WL	OSW	High alpha	Low alpha	Alpha
3 s	0.5 s	86.39	84.15	85.77
	1 s	84.7	82.32	84.53
	2 s	82.57	80.75	83.21
4 s	0.5 s	78.97	78.2	78.46
	1 s	83.86	81.98	83.68
	2 s	81.04	79.31	82.06

### The most significant feature

**Fig. 6:**
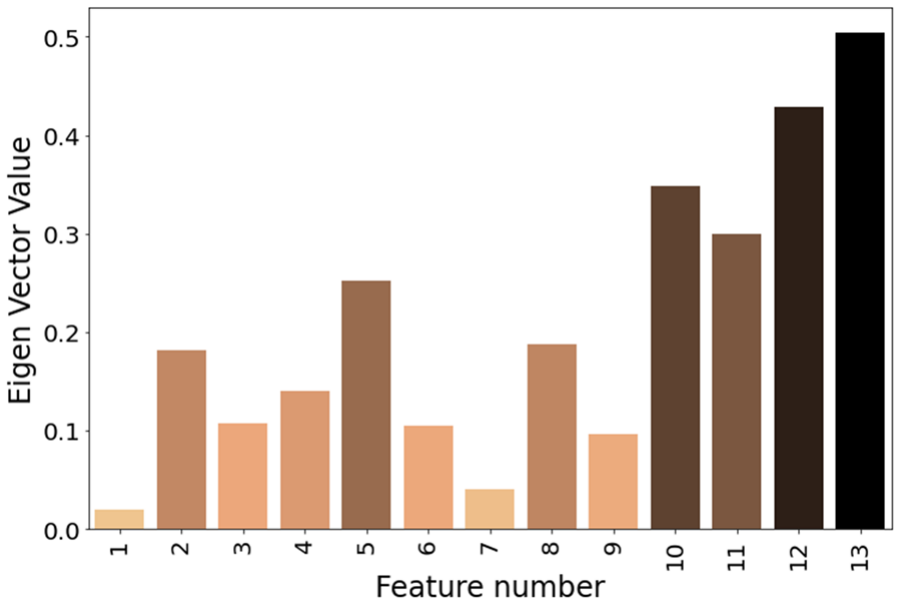
Eigen vector value for each 13 features

Based on the PCA results presented above, we can determine the most significant value by analyzing the eigenvectors of the new space matrix. This can be achieved by sorting the eigenvector values in descending order for each principal component (PC). The magnitude of the values associated with the eigenvectors indicates the significance of each feature. Therefore, a larger magnitude signifies greater significance, as illustrated in Fig. 6.

Figure 6 displays the ranked eigenvector values for all 13 extracted features. This figure was constructed using the data from Fig. 5. Figure 5 provides information on the variance, showing the cumulative contribution from each principal component (PC). The variance values in Fig. 5 help quantify the data variance explained by each PC. A higher variance value indicates a greater amount of variance captured by the data. In our experiment, we set the cumulative variance threshold at 95% [42, 43].

Based on Fig. 6, we can infer that the first six features, namely F13 (amplitude max), F12 (zero crossing), F10 (differential entropy), F11 (band power), F5 (Shannon entropy), and F8 (Hjorth activity), contribute to the classification of the relaxed state. Sanei identified a relationship between the alpha band rhythm and alpha band amplitude while investigating cases of anxiety and sleep disorders [10]. He observed that maximum amplitude is highly correlated with significant features. Similarly, Pane found that differential entropy can serve as a reliable feature for emotion recognition [44]. In Teplan’s research, an increase in alpha band power was noted as an indicator for classifying the relaxed state [19]. Additionally, features such as Shannon entropy, Hjorth activity, and zero crossing are commonly utilized in emotion recognition analyses to demonstrate the complexity of random EEG signals [20, 45].

### Relax acquisition based on time and frequency domains

The Alpha band acts as a marker for identifying the relaxed state. Given the complexity of the alpha band and the individual variations in experiencing relaxation, we proposed the ERA method. ERA aim to identify the exact band and moment when the relaxed state begins. Feature extraction utilizes data from previous steps, employing optimal parameters such as WL, OSW, and selected PC values. The optimal scenario can reach with WL = 3 s, OSW = 0.5 s and 6 PC respectively. To simplify the prediction of the relaxed state, the data is separated into 4-period groups ranges as explained in Fig. 3.

**Fig. 7:**
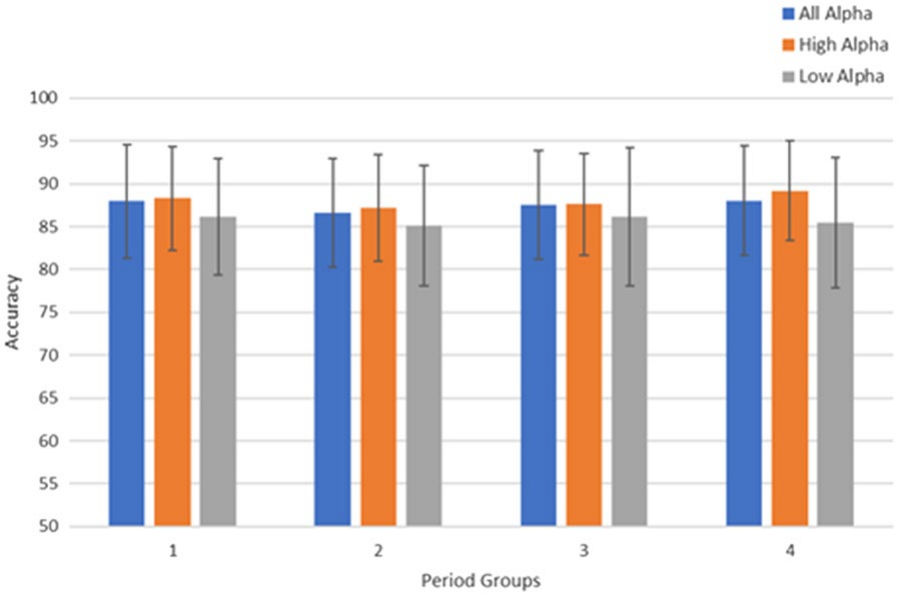
Accuracy and error bar performance of all alpha band, high alpha band and low alpha band in 4-period groups

**Fig. 8:**
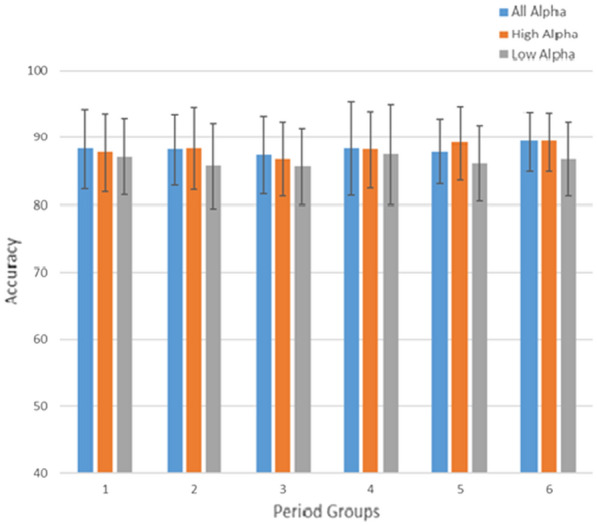
Accuracy and error bar performance of all alpha band, high alpha band and low alpha band in 6-period groups

Figure 7 shows the experimental result. In this figure, the High Alpha show as most optimal accuracy, which is 89.1%. Furthermore, the most optimal accuracy of High Alpha was achieved in the last period grouping, in the 4th period grouping. By observing Figure 7, we can see that the error bar in the High Alpha is stable on all periods with the smallest error bar compared to the Low Alpha or the All Alpha. This experiment shows that the subjects begin to feel relaxed at the end of the given stimulation time.

Figures 7 and 8 provide the information regarding the accuracy and error bar of the relaxed versus sad using the KNN classifier bar. We created two time separation scenarios by grouping the Low Alpha and the High Alpha into 4-period and 6-period groups, with WL 3 s and OSW 0.5 s. Both of 4-period and 6-period groups have different time sampling range, 4-period groups is concist of sampling range: 1st period group (384–2304), 2nd period group (2304–4224), 3rd period group (4224–6144), and 4th period group (6144–8064). While 6-period groups configuration with sampling range 1st period group (384–1.664), 2nd period group (1.664–2.944), 3rd period group (2.944–4.224), 4th period group (4.224–5.504), 5th period group (5.504–6.784) and 6th period group (6.784–8.064).

Comparisons between the 4-period and 6-period groups are illustrated in Figs. 7 and 8. From Fig. 7, we observe that the All Alpha, High Alpha, and Low Alpha categories exhibit better performance in the final 4th group. However, in Fig. 8, this pattern shifts with the 6-period groups configuration. The 5th and 6th periods demonstrate superior performance compared to the other period groups. Both accuracy and error bars show consistent trends across these final period groups. Upon closer examination of the time sampling range configurations between the 4-period and 6-period groups, there is an overlap in the data. Analyzing the data from both groupings, we identified that the relaxed state occurs during the final segment of each period. In the 4-period groups, the relaxed state was observed between 6.144–8.064 (the last segment). Conversely, in the 6-period groups, the relaxed state manifests in the 5.504–6.784 and 6.784–8.064 intervals (the last two segments). Thus, we can conclude that the relaxed state consistently occurs within the time window of 6.144–8.064.

## Discussion

**Fig. 9:**
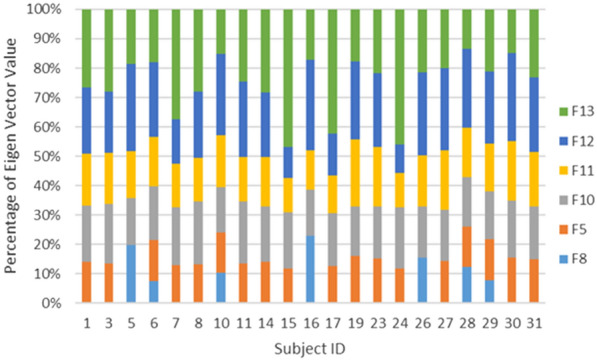
The Eigen vector value from first 6 PC performance of selected subjects in WL=3 s and OSW 0.5 s on the 4th period group

ERA, our proposed method, stands out due to its capacity for feature reduction, achieved through Principal Component Analysis (PCA). It is is achieved through Principal Component Analysis (PCA) computation on each principal component (PC), resulting in six the most significant features. To confirm the importance of these six features, we performed individualized calculations for each subject to ensure fair comparison across all data. Figure 9 presents detailed information on all six features across the selected subjects. This data calculated by utilizing WL 3 s and OSW 0.5 s through High Alpha by observing this figure we can see that significant feature is consistent on 6 PC. Out of 21 subject data in Fig. 9, there are only 5 significant features (F13, F12, F11, F10 and F5). Meanwhile, there are anomalies for three subject with index S5, S16, and S26, their data is lack of F5 feature, and F8 is dominant on those subject. In addition, F13, F12, F11, F10 and F5 can be classified as significant features and dominant in all subjects.

**Fig. 10:**
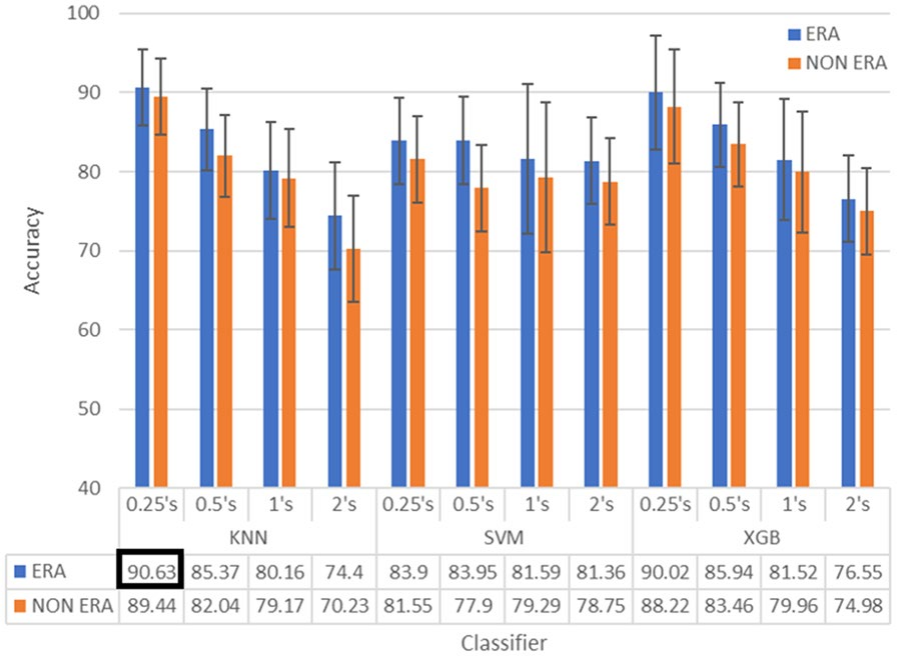
Accuracy comparison performance between ERA and Non ERA on KNN, SVM and XGB classifier

In order to verify the results of the experiments, we complement the experiments by comparing the performance of ERA with non-ERA. This comparison will use a new scenario, by extending the data with WL = 3 s and OSW = 0.25 s, as well as using three classifiers (KNN, SVM and XGB). This comparison method can be seen in Fig. 10, performance of ERA is presented with blue bar and performance of non-ERA with red bar. Based on Fig. 10 we can observe that the performance of ERA is better than the Non ERA performance. This trend is stable through all WL, OSW and classifiers. All classifiers respond positively to this scenario, resulting in better performance. While comparing with other scenarios, we can conclude that small WL and OSW result in more effective accuracy. The Error bar data supported our claim, by showing that the ERA performance is better than non-ERA. This can be obtained by using KNN and XGB as classifiers, both classifiers reach the most optimal performance with a 90% accuracy. However, the error bar of KNN is better than that of the XGB. This supporting our claim that the KNN is perform better than the XGB.

To show the difference between our proposed method and the existing research, we would like to compare our applied method with another proposed method. To provide equal comparison, we use reference another research that utilizes the same dataset, and also research emotional with binary classification by utilizing Alpha sub-band approach. Table 4 summarizes previous research studies using the same dataset. Our proposed method using ERA gained more stable accuracy in multiple classifier machine learning with accuracy over 80%. Meanwhile, the studies conducted by Pane, Lestari, Farashi and Zhang have accuracy below 90%.

**Table 4:** ERA performance comparison with another approach on the same DEAP dataset

Authors	Method	Band	Class	Acc (%)
Pane et al. [46]	CWT	$\alpha$, $\alpha$ sub-band	Relaxed/sad	Low $\alpha$: 81.69 High $\alpha$: 80.14
Lestari et al [4]	Kmeans	$\alpha$	Relaxed/angry	$\alpha$: 67.2
Farashi et al. [47]	Spanning tree	$\alpha$ sub-band	Relaxed/sad	Low $\alpha$: 88.75 High $\alpha$: 81.56
Zhang et al. [48]	Autoregressive	$\alpha$	Relaxed/sad	$\alpha$: 77.75
Our proposed method	ERA	$\alpha$, $\alpha$sub-band	Relaxed/sad	High $\alpha$: 90.63

Our proposed method is superior to other previous relaxed state classification research shown by Table 4. Because our method could classify relaxed state based on less data, this can be obtained by alpha band separation. And with more efficient by reducing dimension by using only 4th period group. This result can be used as a basis for detecting the relaxed state in specific time sampling.

However, during our experiment, we encountered anomalies in the pattern of High Alpha and Low Alpha. We found the relaxed state is associated with a significant value of Low Alpha. Remarkably, our experiment revealed the opposite pattern: The relaxed state exhibited a notable increase in High Alpha activity. To address this issue, we do follow-up discussions with neurologists and experts in EEG-based emotion recognition. Through these discussions, we identified a potential cause for this anomaly: the simulation procedure used during data collection. The dataset was collected from trials in which participants were induced into a relaxed state by watching videos. While this method may yield effective results, it also has its drawbacks. Participants had to stay awake and keep their eyes open while watching videos, starting from the baseline phase and continuing through the stimulus phase. On the contrary, other research suggests that a relaxed state can be effectively induced by having participants close their eyes.

## Conclusion

In this study, we utilized the DEAP dataset to investigate the identification of relaxed states from EEG data. By categorizing observations within the alpha band into two sub-bands (Low Alpha and High Alpha), we developed the Effective Relax Acquisition (ERA) method as a primary tool for classifying relaxed states in EEG recordings. ERA distinguishes itself from similar methods through its dimension reduction capability, which enhances its efficiency and practicality. The essence of ERA involves time and frequency transformations, complemented by PCA for feature selection based on eigenvector values. Our research findings illustrate ERA’s effectiveness in identifying relaxed states, especially notable in the high alpha sub-band during the fourth-period segmentation. To advance future research, we recommend applying and evaluating ERA on diverse datasets, especially those involving emotional EEG data, to enhance the precision of relaxed state identification.

## Data Availability

This experiment uses the DEAP EEG dataset with the emotional label to classify relaxed state conditions. DEAP dataset can be accessed online through  http://www.eecs.qmul.ac.uk/, and an on-demand request EULA (End User License Agreement) is required before accessing this data.
